# Transmission of Severe Acute Respiratory Syndrome Coronavirus 2 via Close Contact and Respiratory Droplets Among Human Angiotensin-Converting Enzyme 2 Mice

**DOI:** 10.1093/infdis/jiaa281

**Published:** 2020-05-23

**Authors:** Linlin Bao, Hong Gao, Wei Deng, Qi Lv, Haisheng Yu, Mingya Liu, Pin Yu, Jiangning Liu, Yajin Qu, Shuran Gong, Kaili Lin, Feifei Qi, Yanfeng Xu, Fengli Li, Chong Xiao, Jing Xue, Zhiqi Song, Zhiguang Xiang, Guanpeng Wang, Shunyi Wang, Xing Liu, Wenjie Zhao, Yunlin Han, Qiang Wei, Chuan Qin

**Affiliations:** NHC Key Laboratory of Human Disease Comparative Medicine, Beijing Key Laboratory for Animal Models of Emerging and Remerging Infectious Diseases, Institute of Laboratory Animal Science, Chinese Academy of Medical Sciences and Comparative Medicine Center, Peking Union Medical College, Beijing, China

**Keywords:** SARS-COV-2, transmission routes, close contact, respiratory droplets, hACE2 transgenic mice, aerosol

## Abstract

We simulated 3 transmission modes, including close-contact, respiratory droplets and aerosol routes, in the laboratory. Severe acute respiratory syndrome coronavirus 2 (SARS-CoV-2) can be highly transmitted among naive human angiotensin-converting enzyme 2 (hACE2) mice via close contact because 7 of 13 naive hACE2 mice were SARS-CoV-2 antibody seropositive 14 days after being introduced into the same cage with 3 infected-hACE2 mice. For respiratory droplets, SARS-CoV-2 antibodies from 3 of 10 naive hACE2 mice showed seropositivity 14 days after introduction into the same cage with 3 infected-hACE2 mice, separated by grids. In addition, hACE2 mice cannot be experimentally infected via aerosol inoculation until continued up to 25 minutes with high viral concentrations.

## Teaser

Severe acute respiratory syndrome coronavirus 2 can be highly transmitted among naive angiotensin-converting enzyme 2 mice via close-contact spreading by respiratory droplets. However, these mice cannot be experimentally infected through aerosol inoculation unless it is continued for 25 minutes with a high viral concentration.

**Figure 1.**
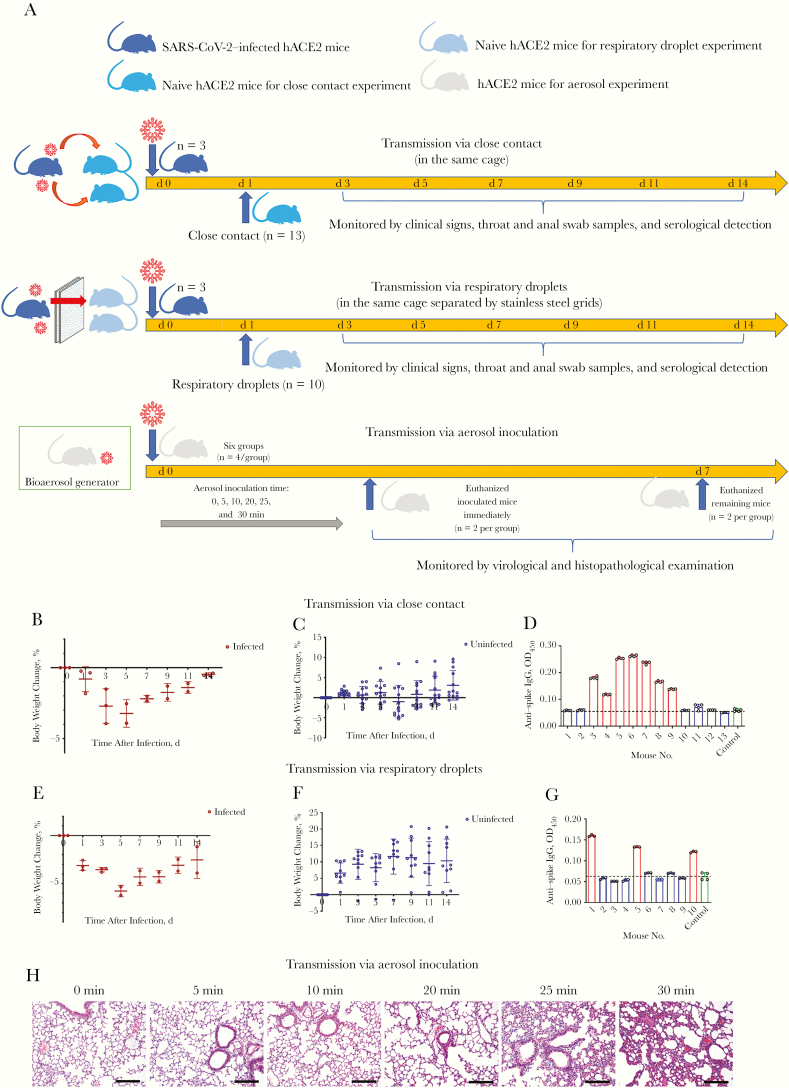
Transmission of severe acute respiratory syndrome coronavirus 2 (SARS-CoV-2) among human angiotensin-converting enzyme 2 (hACE2) transgenic mice via different routes. *A,* Graphic outline of experimental design and sample collection. *B,* The changes in body weight in the 3 hACE2 mice intranasally inoculated with 5 × 10^5^ median tissue-culture infectious dose (TCID_50_) of SARS-CoV-2 used to measure close-contact transmission. *C,* Changes in body weight in the 13 naive hACE2 mice after they were introduced into the same cage with infected mice. *D,* Reactivity of the serum samples from the 13 cohoused mice with SARS-CoV-2 antigens. *E,* Changes in body weight in the other 3 hACE2 mice intranasally inoculated with 5 × 10^5^ TCID_50_ of SARS-CoV-2 used to measure the respiratory droplets transmission. *F,* Changes in body weight in the 10 naive hACE2 mice after introduced into the same cage separated by 2 layers of stainless-steel grids with the infected mice. *G,* Reactivity of the serum samples from the 10 mice for respiratory droplets experiment with SARS-CoV-2 antigens. *H,* Histopathological observation of the hACE2 mice inoculated with aerosol for different durations. Abbreviations: IgG, immunoglobulin G; OD_450_, optical density at 450 nm.

Since December 2019, an outbreak of atypical pneumonia caused by severe acute respiratory syndrome coronavirus 2 (SARS-CoV-2) in Wuhan, China, has become a public health emergency of international concern, as declared by the World Health Organization. The human-to-human transmission of SARS-CoV-2 is the main route of the widespread outbreak, underscoring the necessity of understanding its transmissibility. However, the transmission routes of SARS-CoV-2 with laboratory confirmation have not been documented, and it remains unclear how SARS-CoV-2 is widespread among populations. We have established an angiotensin-converting enzyme 2 (hACE2) transgenic mice model of SARS-CoV-2 infection via intranasal inoculation for epidemic response and directly proved that SARS-CoV-2 could be efficiently transmitted through the respiratory tract [1]. Based on this hACE2 mice model of SARS-CoV-2 infection, the potential transmission routes via close-contact or airborne transmission, including respiratory droplets and aerosol inoculation, were evaluated based on clinical signs, virus replication detection, and serological or histopathological examination, to provide significant data aimed at preventing the spread of SARS-CoV-2 (Figure 1A).

## METHODS

### Ethics Statement

Murine studies were performed in an animal biosafety level 3 facility with high-efficiency particulate air (HEPA)–filtered isolators. All procedures in this experiment involving animals were reviewed and authorized by the Institutional Animal Care and Use Committee of the Institute of Laboratory Animal Science (ILAS), Peking Union Medical College (PUMC) (no. BLL20001).

### Viruses and Cells

The SARS-CoV-2 assigned as SARS-CoV-2/WH-09/human/2020/CHN was isolated by the ILAS, PUMC. Vero cells were prepared for the reproduction of SARS-CoV-2 stocks. The cell line was incubated with Dulbecco’s modified Eagle medium (Invitrogen) complemented with 10% fetal bovine serum, 100 µg/mL streptomycin, and 100 IU/mL penicillin, at 37°C and 5% carbon dioxide_,_. Titers for SARS-CoV-2 were determined using a median tissue culture infectious dose (TCID_50_) assay.

### Animal Experiments

For the animal experiments, specific pathogen-free, 4–6-month- old male and female transgenic hACE2 mice were provided by the ILAS, PUMC. Transgenic mice were generated by microinjection of the mice angiotensin-converting enzyme 2 (ACE2) promoter driving the hACE2 coding sequence into the pronuclei of fertilized ova from Institute of Cancer Research mice, and hACE2 integrated was then identified by means of polymerase chain reaction, as described elsewhere [2]. After intraperitoneal administration of anesthesia with 2.5% tribromoethanol at 0.02 mL/g, the hACE2 mice were inoculated via the intranasal route with SARS-CoV-2 stock virus, at a dose of 10^5^ TCID_50_. The infected animals were continuously observed daily to record body weights, clinical symptoms, and death. The throat and anal swab samples were collected on 0, 3, 5, 7, and 14 days after inoculation.

### Statistical Analyses

All data were analyzed using GraphPad Prism 8.0 software. Statistically significant differences were determined using unpaired *t* tests.

## RESULTS

### High Risk of Transmission Between Naive and Infected hACE2 Mice in Close Contact

Three specific pathogen-free, 4–6-month-old hACE2 mice were intranasally inoculated with 1 × 10^5^ TCID_50_ of SARS-CoV-2 and placed in a single special-transmission cage (43 × 28 × 18 cm). On day 1 after inoculation, 13 naive-hACE2 mice were introduced with the inoculated mice (Figure 1B) to assess the effect of close-contact transmission. The mice were numbered from 1 to 13. On day 3 after inoculation, 5 of the 13 mice exhibited weight loss (mice 4, 7, 8, 9, and 11); by day 7, weight loss was had occurred in 8 of 13 mice (mice 2–9); the maximal weight loss observed in a single mouse was 5.28% (Figure 1C). 

Although throat and anal swab samples were collected on days 3, 5, 7, and 14 after inoculation from each mouse, the viral load was detectable in only 3 of the 13 throat swab samples (viral load, 10^2.93^, 10^2.91^, and 10^2.95^ copies/mL, in mice 5, 6, and 7, respectively) and 1 of the 13 anal swab samples on day 5 (viral load, 10^1.07^ copies/mL in mouse 7). All of the serum samples were collected on day 14 to detect the presence of immunoglobulin G antibodies reactive with SARS-CoV-2 antigens (the optical density at 450 nm value for serum samples was considered positive result when it was at least twice that of the negative control). The mice with detectable viral loads in the swab samples also showed SARS-CoV-2 antibodies. In total, 7 of the 13 mice (mice 3–9) were infected after direct or close contact, based on the serological analyses, consistent with the individual data for weight loss in Table 1 (Figure 1D).

**Table 1.** Detection of Viable Severe Acute Respiratory Syndrome Coronavirus 2 After Inoculation of Mice via Close Contact or Respiratory Droplets

Route of administration	Time After Inoculation, d	Clinical Sign, No./Total Observed			Viral RNA Detected in Swab Samples, No./Total		SARS-CoV-2–Specific IgG Antibodies^b^
		Weight Loss^a^	Asthenia	Death	Throat	Anal	
Close contact	0	0/13	0/13	0/13	0/10	0/10	NE
	1	0/13	0/13	0/13	0/10	0/10	NE
	3	5/13 (6.77%)	0/13	0/13	0/13	0/13	NE
	5	4/13 (7.72%)	0/13	0/13	3/13^c^	1/13^d^	NE
	7	8/13 (15.45%)	0/13	0/13	0/13	0/13	NE
	9	6/13 (7.86%)	0/13	0/13	0/13	0/13	NE
	11	5/13 (4.21%)	0/13	0/13	0/13	0/13	NE
	14	2/13 (1.97%)	0/13	0/13	0/13	0/13	7/13
Respiratory droplets							
	0	0/10	0/10	0/10	0/10	0/10	NE
	1	0/10	0/10	0/10	0/10	0/10	NE
	3	1/10 (6.02%)	0/10	0/10	0/10	0/10	NE
	5	1/10 (4.25%)	0/10	0/10	0/10	0/10	NE
	7	1/10 (5.23%)	0/10	0/10	0/10	0/10	NE
	9	0/10	0/10	0/10	0/10	0/10	NE
	11	0/10	0/10	0/10	0/10	0/10	NE
	14	0/10	0/10	0/10	0/10	0/10	3/10

Abbreviations: IgG, immunoglobulin G; NE, not examined; SARS-CoV-2, severe acute respiratory syndrome coronavirus 2.

^a^Parenthetical percentages in weight loss column represent the percentages of mean maximum weight loss after inoculation

^b^Data were recorded as mean optical density values, based on ≥4 tests for each sample. The cutoff value is twice the mean value of the negative control (the mean for 4 uninfected mice.), 0.059 in these experiments.

^c^Viral loads: 2.93, 2.91, and 2.95 log_10_ RNA copies/mL.

^d^Viral load: 1.07 log_10_ RNA copies/mL.

### Respiratory Droplets as Important Transmission Route

Another 3 specific pathogen-free, 4–6-month-old hACE2 mice were intranasally inoculated with 1 × 10^5^ TCID_50_ of SARS-CoV-2. On day 1 after inoculation, they were placed in another special-transmission cage for an experiment with respiratory droplets. This cage was designed to prevent any direct contact and allow only airflow between the inoculated and the neighboring naive mice. The transmission cage was specifically designed to enable transmission experiments to be conducted in an individual ventilated cages (43 × 28 × 18 cm) in an animal biosafety level 3 facility (Supplementary Figure 1). The cage was separated by 2-layer stainless steel grids, with 0.5-cm^2^ openings spaced 1.5 cm apart, to facilitate air flow between the 2 sides. The distance between the 2 grids was 2 cm. The outlet airflow is HEPA filtered to prevent continuous circulation of SARS-CoV-2 particles and to prevent cross-contamination. 

The 3 infected-hACE2 mice (Figure 1E) were separated by 2-layer stainless steel grids from 10 naive-hACE2 mice to model airborne transmission only via respiratory droplets [3]. Only 1 of the 10 mice exhibited weight loss on the day 3 after inoculation, and the other 9 showed weight gain; the maximal increase observed in 1 mouse was 1.92% (Figure 1F). Throat and anal swab samples were collected from the inoculated animals on days 3, 5, 7, and 14 after inoculation, and no viral RNA was detected in any of the samples. It is noteworthy that 3 of the 10 serum samples collected on day 14 reacted with the SARS-CoV-2 antigens in serological analyses, including serum from the mouse that lost weight. Therefore, we confirmed that 3 of the 10 mice were infected via respiratory droplets (an optical density at 450 nm value at least twice the mean of the negative control was regarded as a positive result) (Figure 1G).

### Aerosol Inoculation of hACE2 Mice Ineffective Unless Continued for 25 Minutes with High Viral Concentrations

Finally, the transmissibility of SARS-CoV-2 in transgenic hACE2 mice via aerosol infection was also evaluated under experimental conditions by means of virological and histopathological examinations. Specific pathogen-free, 4-6-month-old male and female hACE2 mice were divided into 6 groups (n = 4 per group) and were inoculated via the intranasal route at a dose of 2 × 10^6^ TCID_50_/mL, using a bioaerosol generator (In-Tox Products; Moriarty). The exposure dosage of the virus was 36 TCID_50_/min, and the exposure times for the groups of hACE2 mice were 0, 5, 10, 20, 25, and 30 minutes. Two mice in each group were euthanized immediately after exposure to analyze the pulmonary viral load. The results demonstrated that viruses could be detected in the lungs only after exposure of up to 25 minutes, and the mean viral loads were 10^2.07^ (10^2.08^ and 10^2.06^ in the 2 euthanized mice) and 10^2.11^ (10^2.13^ and 10^2.09^) RNA copies/mL after exposure for 25 and 30 minutes, respectively (Table 2). 

**Table 2.** Detection of Viable Severe Acute Respiratory Syndrome Coronavirus 2 After Inoculation of Mice via Aerosol

Viral RNA or Pathological Changes	Duration of Aerosol, min					
	0	5	10	20	25	30
Viral RNA detected, no. of samples/total						
0 d	0/2	0/2	0/2	0/2	2/2^a^	2/2^b^
7 d	0/2	0/2	0/2	0/2	2/2^c^	2/2^d^
Pathological changes						
7 d	0/2e	0/2e	0/2e	0/2e	**+**f	**+**f

^**a**^Viral loads: 2.08 and 2.06 log_10_ RNA copies/mL.

^b^Viral loads: 2.13 and 2.09 log_10_ RNA copies/mL.

^c^Viral loads: 3.68 and 3.70 log_10_ RNA copies/mL.

^d^Viral loads: 3.70 and 3.72 log_10_ RNA copies/mL.

^e^there was no significant pathological change in the lung.

^f^there was mild interstitial pneumonia in the lung.

The remaining 2 hACE2 mice in each group were euthanized on day 7 after inoculation. After necropsy, the lungs were collected for viral load detection and routine histopathological observation. Consistent with the data on the first day, the last 2 groups (with exposure for 25 and 30 minutes) displayed detectable viral replication in the lungs, at mean values of 10^3.69^ (10^3.68^ and 10^3.70^) and 10^3.71^ (10^3.70^ and 10^3.72^) RNA copies/mL, respectively (Table 2). Mild interstitial pneumonia was observed in these 2 groups, including mild, focal thickened alveolar septum and infiltration of mainly lymphocytes and mononuclear cells around the bronchioles and blood vessels, without alveolar exudation (Figure 1H). Compared with the lung tissues in the control group (0 minutes), those in the other 3 groups (5, 10, and 20 minutes) had no significant lesions. Therefore, the hACE2 mice cannot be experimentally infected via aerosol inoculation until exposure is continued up to 25 minutes with high viral concentrations.

## Discussion

Human-to-human transmission via close contact is believed to be the primary means of transmission for SARS infections. Transport of the virus via liquid droplets released from an infected person through respiration, coughing, or sneezing is considered the source, especially in a closed and poorly ventilated environment [4]. Our research emphasized that SARS-CoV-2 can be experimentally transmitted among hACE2 mice by close contact, through respiratory droplets, but is hardly transmitted through aerosol inoculation. Close contact is an accumulation mode for various transmission routes, consistent with the result that this route is more efficient than other transmission routes. To our knowledge, the current study provides the first laboratory-based evidence regarding potential infective routes of human-to-human transmission of SARS-CoV-2, providing significant data to prevent the human pandemic of SARS-CoV-2.

## Supplementary Data

Supplementary materials are available at *The Journal of Infectious Diseases* online. Consisting of data provided by the authors to benefit the reader, the posted materials are not copyedited and are the sole responsibility of the authors, so questions or comments should be addressed to the corresponding author.

jiaa281_suppl_Supplementary_Figure_1

jiaa281_suppl_Supplementary_Material

## Notes

***Author contributions.*** Conceptualization: C. Q. Methodology: L. B., H. G., W. D., Q. L., H. Y., M. L. and C. Q. Investigation: All authors. Writing (original draft): J. X. and Z. S. Writing (review and editing): C. Q. Funding Acquisition: L. B. and C. Q. Resources and supervision: C. Q.

***Financial support.*** This work was supported by the National Key Research and Development Project of China (grant 2016YFD0500304), the CAMS initiative for Innovative Medicine of China (grant 2016-I2M-2–006), and National Mega projects of China for Major Infectious Diseases (grant 2017ZX10304402).

***Potential conflicts of interest.*** All authors: No reported conflicts. All authors have submitted the ICMJE Form for Disclosure of Potential Conflicts of Interest. Conflicts that the editors consider relevant to the content of the manuscript have been disclosed.
