# Supplementary figures and images for: Transmission of Severe Acute Respiratory Syndrome Coronavirus 2 via Close Contact and Respiratory Droplets Among Human Angiotensin-Converting Enzyme 2 Mice
Source: J Infect Dis. 2020 May 23;222(4):551–5. doi: 10.1093/infdis/jiaa281 (PMC7313959; doi:10.1093/infdis/jiaa281)

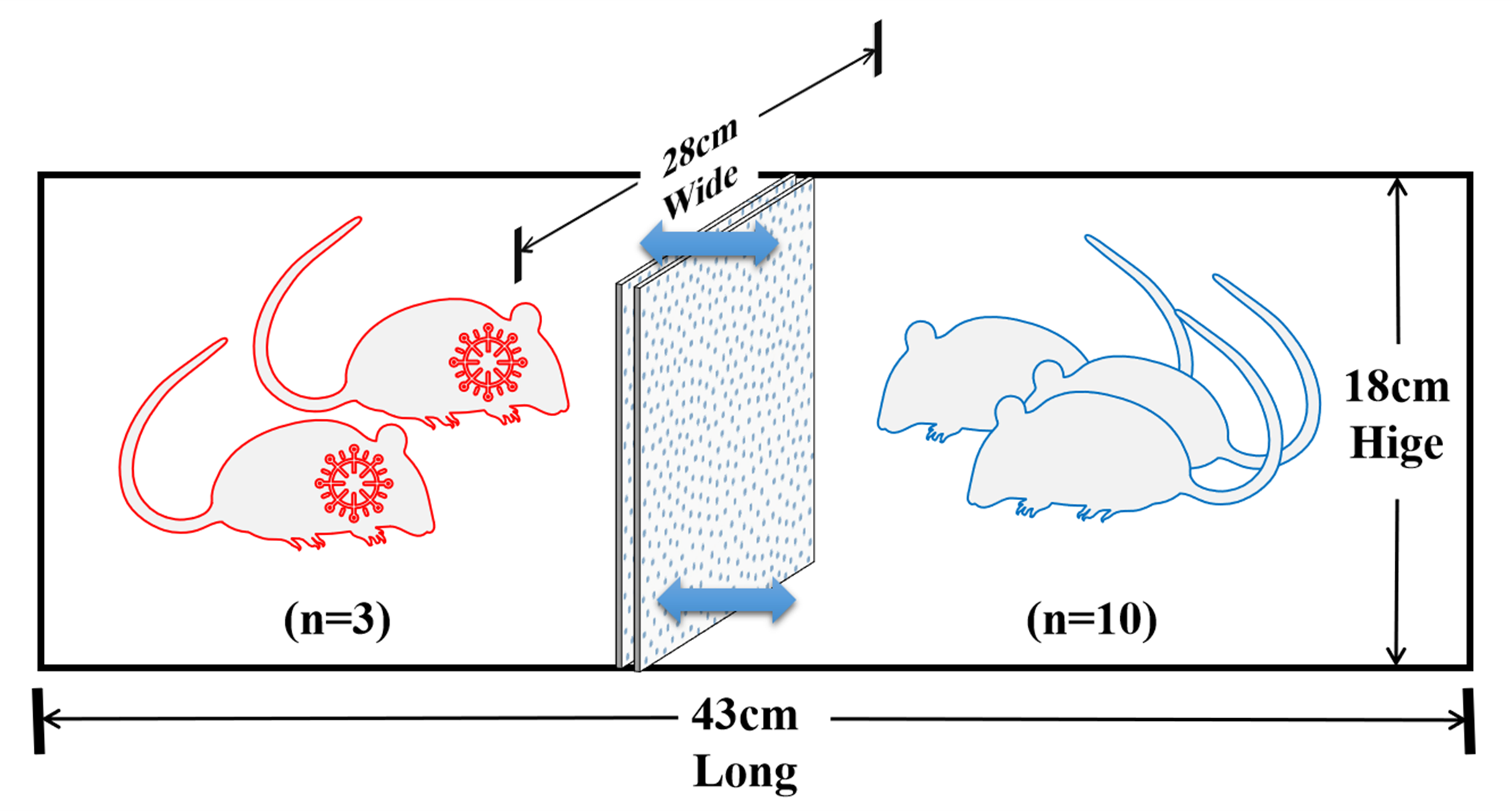

Supplement: jiaa281_suppl_Supplementary_Figure_1 [file jiaa281_suppl_supplementary_figure_1.png]
