# Supplementary material for: Transmission of Severe Acute Respiratory Syndrome Coronavirus 2 via Close Contact and Respiratory Droplets Among Human Angiotensin-Converting Enzyme 2 Mice
Source: J Infect Dis. 2020 May 23;222(4):551–5. doi: 10.1093/infdis/jiaa281 (PMC7313959; doi:10.1093/infdis/jiaa281)
Supplement: jiaa281_suppl_Supplementary_Material [file jiaa281_suppl_supplementary_material.docx]

**Supplementary data**

**Methods**

**Preparation of homogenate supernatant**

Tissue homogenates (1 g/mL) were prepared by homogenizing perfused tissues using an electric homogenizer for 2 min 30 s in DMEM. At 4°C, the homogenates were centrifuged at 3,000 rpm for 10 min. For viral titer and viral loads detection, the supernatant was collected and stored at −80°C.

**The tissue culture infectious dose 50 (TCID_50_) assay**

The TCID_50_ assay were performed as previous reports [1]. Briefly, to measure the titers of SARS-CoV-2, 10-fold serial dilutions of the viruses were used to inoculate Vero cell monolayers in DMEM containing 2% fetal bovine serum at 37°C for 72 hours. After 72 hours of infection, the cytopathic effect (CPE) was observed, and the tissue culture infectious dose 50 (TCID_50_) values were calculated by the Reed and Muench method [2].

**RNA extraction and qRT-PCR**

Total RNA was extracted and detected as described previously [3]. The primer sequences used for RT-PCR are targeted against the envelope (E) gene of SARS-CoV-2 and are as follows: forward primer sequence 5’-TCGTTTCGGAAGAGACAGGT-3’ and reverse primer sequence 5’-GCGCAGTAAGGATGGCTAGT-3’. The PCR products were verified with sequencing using the dideoxy method on an ABI 3730 DNA sequencer (Applied Biosystems, CA, USA). The SYBR green real-time PCR standard curve was generated with serial 10-fold dilutions of recombinant plasmid with a known copy number (from 1.47 x 10^9^ to 1.47 x 10^1^ copies/μL). These dilutions were tested and used as quantification standards to construct the standard curve by plotting the plasmid copy number against the corresponding threshold cycle values (CT). Results are expressed as log10 numbers of genome equivalent copies (GEC) per mL of sample.

**ELISA analyses**

The specific IgG against SARS-CoV from mice was determined using enzyme-linked immunosorbent assay (ELISA). The Spike 1 protein of SARS-CoV-2 (0.1 μg/100 μL, Sino Biological, 40591-V08H) was prepared to coat the 96-well plates; the sera were diluted at 1:100 and added to each well; triple wells were set for each sample and then incubated at 37°C for 30 min, then incubated by the goat anti-mouse secondary antibodies conjugated with HRP (ZB-2305, zhongshan,1:10,000 dilution), and incubated at room temperature for 30 min. The reaction was developed with TMB substrate, and optical densities were determined at 450 nm (Metertech960 enzyme marker with 450 nm wavelength).

**The aerosol experiment**

The aerosol was generated using the In-Tox Products (Moriarty, NM, USA). The virus stock at a dosage of 2×10^6^ TCID_50_/mL was used to generate aerosol by In-Tox Products (Moriarty, NM, USA). The exposure dose of the virus was 36 TCID_50_/min. The exposure time for hACE2 mice were 0 min, 5 min, 10 min, 20 min, 25 min, and 30 min, respectively.

The exposure dose is generally defined by the following equation (1):

| $Emin=\frac{Vmin}{Va+Vb} \cdot\frac{C\cdot V}{T}$ | （1） |
| --- | --- |

with Emin being the inhaled virus per minute of exposure (TCID_50_/min), Vmin is the minute ventilation of an animal (mL), Va is the volume of atomization flow per minute (mL), Vb is the volume of dilute flow per minute (mL), T is the exposure time (min), V is the volume of the virus stock consumed by the aerosol generator (mL), C is The virus concentration of virus stock (TCID_50_/mL).

In our calculating process, Vmin = 40mL, Va = 600mL, Vb = 18300mL, T = 30min, V = 0.25mL, C = 1×10^6^ TCID_50_/mL. Thus, we obtain the value of Emin which was equal to 18.

$Emin=\frac{\mathrm{Vmin}}{Va+Vb} \cdot\frac{C\cdot V}{T}$ = $\frac{40}{600+18300}\cdot\frac{{2\times10}^{6}\cdot0.25}{30}\approx36$ TCID_50_/min

**Pathological examination**

Autopsies were prepared in the animal biosafety level 3 (ABSL3) laboratory. The primary organs were grossly observed and then fixed in 10% buffered formalin solution; paraffin sections (3-4 µm in thickness) were prepared routinely. Hematoxylin and Eosin stain was used to identify histopathological changes in all the organs. The histopathology of the lung tissue was observed using light microscopy.

**References:**

1. Bao L, Deng W, Huang B, et al. The Pathogenicity of SARS-CoV-2 in hACE2 Transgenic Mice. bioRxiv 2020

2. Reed LJ MH. A simple method of estimating fifty percent endpoints. Am J Hyg 1938; 27:493-7.

3. Gong S, Qi F, Li F, et al. Human-Derived Th005 (H7N9) Exhibits Extremely High Replication in the Lungs of Ferrets and is Highly Pathogenic in Chickens. Viruses 2019.

**Supplementary Figure 1. A schematic diagram of the special transmission cage for respiratory droplets experiment.** The transmission cage was specifically designed to allow transmission experiments to be conducted in an individual ventilated cages (43×28×18 cm) in an animal biosafety level 3 facility. The cage was separated by double layer-stainless steel grids, with a grid size of 0.8 cm^2^, 1.5 cm apart, to facilitate air flow between both sides. The distance between the two grids is 2cm. The outlet airflow is high-efficiency particulate air (HEPA) filtered to prevent continuous circulation of SARS-CoV-2 particles and to prevent cross-contamination. Arrows in the schematic diagram indicate the airflow direction.
